# Supplementary material for: The impact of genetic adaptation on chimpanzee subspecies differentiation
Source: PLoS Genet. 2019 Nov 25;15(11):e1008485. doi: 10.1371/journal.pgen.1008485 (PMC6901233; doi:10.1371/journal.pgen.1008485)
Supplement: S1 Appendix — (DOCX) [file pgen.1008485.s001.docx]

# S1 Appendix.

## Signed differences in derived allele frequency (*δ*) amongst human populations.

We were interested in comparing the recent adaptive history of chimpanzees and humans. Previously [1] found that that those SNPs with the greatest allele frequency difference between populations of modern humans were enriched for genic variants. Subsequent work presented by [2] and [3] replicated these findings. To present consistent analyses and make more specific comparisons with chimpanzees, we replicated the analyses of signed differences in derived allele frequency (*δ*) in three human populations: Yoruba in Ibadan, Nigeria (YRI); Japanese in Tokyo, Japan (JPT); British in England and Scotland (GBR). We choose JPT and GBR because their pairwise F_ST_ is 0.10 i.e. approximately the same as for eastern and central chimpanzees. The YRI- vs. non-African pairwise comparisons have amongst the largest F_ST_ values of all comparisons among 1000 Genomes populations, ~0.15 (data not shown). By down sampling each population to n =10 or n = 20 individuals, we can also assess the impact of sample size, considering that the range of chimpanzee samples is 10 -19. We used genotype data from the 1000 genomes phase III [4], without any filtering of genotypes. We used the annotated ancestral allele in this same dataset (which are derived themselves from EPO alignments) to polarise derived allele frequencies.

We find that sample size has moderate effects on the determination of *δ* tail bin genic enrichment, except for GBR vs. JPT, which have few SNPs with a large frequency difference, and for which the high resolution afforded with sample n=91 is required to ascertain a significant tail bin enrichment (Figure S1a).

When comparing the 95% confidence intervals of tail bin genic enrichments we find, consistent with previous results, that *δ* tail bin genic enrichments are symmetrical for human populations when ancient DNA information is not incorporated [3]. This finding is consistent across all sample sizes (Figure S1a), suggesting that sample size is not a contributor to the stronger genic enrichment in central vs. eastern chimpanzees.

To further explore asymmetry among the genic enrichment in the two tails of *δ*, we repeat the calculation of tail bin genic enrichment log_2_ ratios as in Main Text Figure 2. In nearly all cases the enrichment is symmetric (Figure S1b). The only significant asymmetry is for an increased genic enrichment in Yoruba vs. Japanese when the sample size is 91. We note that despite being significant, this asymmetry is only half that observed in the comparison between eastern chimpanzees and central chimpanzees. No human comparison thus shows signatures that compare to those between eastern and central chimpanzees.

1. Coop G, Pickrell JK, Novembre J, Kudaravalli S, Li J, Absher D, et al. The role of geography in human adaptation. PLoS Genetics. 2009;5(6):e1000500-e. doi: 10.1371/journal.pgen.1000500.

2. Hernandez RD, Kelley JL, Elyashiv E, Melton SC, Auton A, McVean G, et al. Classic selective sweeps were rare in recent human evolution. Science. 2011;331(6019):920-4. doi: 10.1126/science.1198878.

3. Key FM, Fu Q, Romagne F, Lachmann M, Andres AM. Human adaptation and population differentiation in the light of ancient genomes. Nature Communications. 2016;7:10775-. doi: 10.1038/ncomms10775.

4. Auton A, Brooks LD, Durbin RM, Garrison EP, Kang HM, Korbel JO, et al. A global reference for human genetic variation. Nature. 2015;526(7571):68-74. doi: 10.1038/nature15393.
