# Supplementary material for: The impact of genetic adaptation on chimpanzee subspecies differentiation
Source: PLoS Genet. 2019 Nov 25;15(11):e1008485. doi: 10.1371/journal.pgen.1008485 (PMC6901233; doi:10.1371/journal.pgen.1008485)
Supplement: S2 Appendix — (DOCX) [file pgen.1008485.s002.docx]

# S2 Appendix.

## Estimating the strength of background selection required to explain *δ* bin genic enrichments.

Previously it has been shown that background selection (BGS) can result in genic enrichment in sites with large frequency differences between populations [1-3]. To find the strength of BGS (measured as a B score, a fraction of the expected neutral diversity) that could explain genic enrichments observed in chimpanzees, we simulated 10 million 2kb loci for non-genic (B = 1) and genic regions. For genic regions we used a range of B (1 – 0.8) in 0.025 steps, except between 0.9 – 0.85 for which we used a step size of 0.0125. While the strength of purifying and background selection varies among genes and genomic regions, this global (average) inference allows us to make comparisons at the genome scale. We used the sum of squared differences between simulated and observed genic enrichments for *δ* bins to ascertain which B provides the best fit to observed genic enrichments.

We find that when fitting all *δ* bins for all pairwise *δ,* the best fit is provided by B = 0.888 (S3 Table), which indicates a reduction in neutral diversity levels of 11% in genes when compared with non-genes.

We repeated this exercise using data on the 12 *δ* tail bins alone. Doing so allows us to infer the strength of BGS required to fully explain the genic enrichment in the most highly differentiated SNPs, which likely harbour targets of positive selection. While assuming no influence of positive selection is unrealistic, this allows us to explore whether background selection alone could, in theory, explain our observations. The best fitting B in this case is 0.863, or a 14% reduction in genic diversity levels due to BGS (S3 Table).

In contrast, by excluding the 12 *δ* tail bins, the fit of observed to simulated genic enrichments is less likely to be reduced due to the influence of targets of positive selection. The best fitting B in this case is 0.925 (S3 Table).

Comparing the relative order of magnitudes of the Sum of Squares shows that the worst fit of simulated and observed genic enrichment are seen when attempting to fit all *δ* bins. This is an indication that BGS is not the only force affecting drift and diversity levels in genes, and combined with the observation that the best fit is B=0.925 when *δ* tail bins are excluded suggests positive selection is contributing to the genic enrichments in the *δ* tails.

We also checked if a greater genic enrichment in eastern vs. central chimpanzees is expected given the demographic history of chimpanzees and/or the effects of BGS. For each value of B we modelled above, we also calculated the log_2_ ratio of the eastern and central *δ* tail bin.

No value of B in the range 1 – 0.8 results in an asymmetry in genic enrichment between eastern and central chimpanzees as great as that observed in the genomic data (max B = 0.850, 0.103; observed 0.34). No large asymmetry is generated under the demographic model without BGS (B =1). Both results suggest that no combination of BGS strength can produce the difference in eastern and central *δ* tail bin genic enrichment observed.

1. Coop G, Pickrell JK, Novembre J, Kudaravalli S, Li J, Absher D, et al. The role of geography in human adaptation. PLoS Genetics. 2009;5(6):e1000500-e. doi: 10.1371/journal.pgen.1000500.

2. Hernandez RD, Kelley JL, Elyashiv E, Melton SC, Auton A, McVean G, et al. Classic selective sweeps were rare in recent human evolution. Science. 2011;331(6019):920-4. doi: 10.1126/science.1198878.

3. Key FM, Fu Q, Romagne F, Lachmann M, Andres AM. Human adaptation and population differentiation in the light of ancient genomes. Nature Communications. 2016;7:10775-. doi: 10.1038/ncomms10775.
