# Supplementary material for: The impact of genetic adaptation on chimpanzee subspecies differentiation
Source: PLoS Genet. 2019 Nov 25;15(11):e1008485. doi: 10.1371/journal.pgen.1008485 (PMC6901233; doi:10.1371/journal.pgen.1008485)
Supplement: S3 Appendix — (DOCX) [file pgen.1008485.s003.docx]

# S3 Appendix.

## Evidence for, and explanatory power of, differing strengths of BGS amongst chimpanzees.

We note first the evidence suggesting that background selection varies little among the great apes despite their large differences in *N_e_* [1] and despite the stronger purifying selection in larger *N_e_* subspecies [2]. Background selection is expected to reduce diversity in genic regions more than in non-genic ones by removing variants linked to deleterious alleles, but the action of this type of selection appears independent of *N_e_* [1]. Background selection is instead determined by the distribution of fitness effects for deleterious alleles, which is likely similar among the great apes owing to their generally conserved gene location and function [1]. Further, simulations show that the rate of selective sweeps explains the larger reduction of diversity around genes in species with larger *N_e_* [1]. Thus, the diversity reducing effect of background selection should be the same across all four chimpanzee sub-species. We tested this comparing the levels of scaled neutral diversity (π / divergence to macaque) between chimpanzee sub-species as a function of the distance to the nearest gene (normalized to the lowest diversity seen for each of the sub-species Figure S4a). We confirmed that the relative reduction in neutral variation linked to genes is the same across sub-species (both Figure S4a), and that the nucleotide distance from genes at which neutral diversity reaches equilibrium is also similar. In addition, we find that the average genomic diversity in central, eastern and Nigeria-Cameroon chimpanzees has similar dependency on recombination rate and density of functional features (gene coding and gene untranslated sequences and non-coding conserved elements (Figure S4b) suggesting yet again that background selection is comparable among them. Note however that functional categories appear to be worse predictors of diversity levels in western chimpanzees than the other subspecies (95% CI of the bootstrap distributions of *rho*, the partial *spearmans* correlation controlling from recombination rate, do not overlap). We have not investigated this further, but it is possibly due to the fact that the genetic map is based on a sample of western chimpanzees, is therefore most accurate for this subspecies with the effect of smaller residuals in the regression of diversity on recombination rate.

Lastly, we turn to a population genetic statistical model able to estimate the reduction in neutral diversity due to background selection[3]. Full details for this model are given in Corbett-Detig *et. al.* (2015), but we briefly recapitulate the main points. The effect of BGS is estimated as the population scaled mutation rate (4*N_e_μ*, θ) scaled by a parameter G, that models BGS as a local reduction in *N_e_* with G allowed to vary in windows along the genome in proportion to the per window fraction of functional sites. The effect of selective sweeps or Hitch Hiking (HH) is also estimated as θ divided by the population scaled rate of sweeps, 2*Nv*. Following the implementation of this model by Corbett-Detig *et. al.* (2015) we calculated average neutral diversity in 500 kb windows and used the number of bp in exons for functional density. We ran the compute_gk package on this data to estimate the effects of linked selection, as described by Corbett-Detig *et. al.* (2015). Using this model, we estimated the same reduction in neutral diversity in each chimpanzee subspecies (11% reduction in the highest likelihood model; S5 Table), indicating equivalent levels of background selection among sub-species.

Despite there being no evidence to suggest that there are differences in the strength of BGS between eastern and central chimpanzees, it is useful to determine if such a putative asymmetry in BGS strength could lead to eastern chimpanzees having a greater tail bin genic enrichment than central chimpanzees. To investigate this possibility, we performed simulations of BGS, where the strength of BGS was stronger in eastern chimpanzees (B = 0.825) than in all other chimpanzees (B range: 0.900 – 0.850). We find that while a greater eastern B marginally increases the relative magnitude of eastern chimpanzee *δ* tail bin genic enrichment, none of the simulated ratios are within the 95% of the eastern vs. central *δ* tail bin log_2_ ratio. This further reinforces stronger BGS in eastern than in other chimpanzees would result in differences in *δ* tail bin genic enrichment.

1. Nam K, Munch K, Mailund T, Nater A, Greminger MP, Krützen M, et al. Evidence that the rate of strong selective sweeps increases with population size in the great apes. Proceedings of the National Academy of Sciences. 2017;114(7):1613-8. doi: 10.1073/pnas.1605660114.

2. Bataillon T, Duan J, Hvilsom C, Jin X, Li Y, Skov L, et al. Inference of purifying and positive selection in three subspecies of chimpanzees (Pan troglodytes) from exome sequencing. Genome Biology and Evolution. 2015;7(4):1122-32. doi: 10.1093/gbe/evv058.

3. Corbett-Detig RB, Hartl DL, Sackton TB. Natural Selection Constrains Neutral Diversity across A Wide Range of Species. PLoS Biology. 2015;13(4):e1002112-e. doi: 10.1371/journal.pbio.1002112.
