# Supplementary material for: The impact of genetic adaptation on chimpanzee subspecies differentiation
Source: PLoS Genet. 2019 Nov 25;15(11):e1008485. doi: 10.1371/journal.pgen.1008485 (PMC6901233; doi:10.1371/journal.pgen.1008485)
Supplement: S4 Appendix — (DOCX) [file pgen.1008485.s004.docx]

# S4 Appendix

**Population branch statistics**

In the two-population case (Pop A, Pop B), a “scan” for targets of population selection can be performed by identifying outliers – e.g. the top 5% of sites – in the genome wide distribution of per site pairwise F_ST_ values, if one assumes that these outliers are likely enriched for true targets of positive selection (the empirical distribution could also be compared to simulated values). As pairwise F_ST_ is a summary of the joint site frequency spectrum (SFS) of two populations, these outliers are the sites with the greatest site (allele) frequency difference. If one considers this as an unrooted two population tree (i.e. a straight line), outliers are simply those sites with the longest branch lengths. The problem of course is that there is no directionality to F_ST_, but one assumes that the population with the highest derived allele frequency is the one in which selection has acted.

The Population Branch Statistic (PBS), introduced by [1] in their study that identified *EPAS1* as under selection in Tibetans, extends the pairwise F_ST_ case by the addition of a third population, Pop C. PBS is a function of the three possible pairwise F_ST_ values amongst three populations (AB, AC, and BC). As in the two-population case, there is only one unrooted tree relating three populations, with each population connected to the central node. Therefore, each population can be assigned a unique branch length or PBS value (Figure S11a). The branch length is indicative of the population specific change in allele frequency, and targets of positive selection can be identified as outliers. Thus, PBS overcomes the issue of assigning directionality to allele frequency differences between populations, although with the assumption that selection occurs in one branch only.

We wanted to analyse the joint frequency spectrum of the four chimpanzee subspecies, and used PBS as an inspiration to develop a new statistic, PBSnj. We analyse a simple four population model, with two groups of sister taxa A,B and C,D sharing a common ancestor AB,CD. Split times for AB,CD, A,B and C,D are 0.2, 0.1, 0.1 scaled time units respectively, and population size is 10e^3^ throughout. We performed 2 million simulations of a 2kb locus, with mutation rate = 1.2e^-8^ and recombination rate = 0.96e^-8^, and sampling 50 chromosomes per population. The msms [2] command line used is:

msms 200 1 -t 0.96048 -r 0.768384 -I 4 50 50 50 50 -n 1 1 -n 2 1 -n 3 1 -n 4 1 -en 0.1 2 1 -en 0.1 4 1 -ej 0.1 1 2 -ej 0.1 3 4 -en 0.2 4 1 -ej 0.2 2 4.

We take A as the focal population. There are three possible combinations of F_ST_ values to calculate the branch length leading to population A: ABC, ABD, and ACD, denoted PBS_ABC_ *etc*. We note that in the Tibetan PBS example, populations were chosen so that one was clearly ancestral: Danish is the outgroup to Tibetan and Han. This highlights that while the underlying tree is unrooted and the Tibetan branch represents allele frequency change since their split with Han Chinese, in reality the Danish branch is a compound branch length combining branches leading from the basal Eurasian common ancestor to the Danish and the basal Eurasian common ancestor to the common ancestor of Tibetans and Han Chinese. In this sense, the Danish PBS branch would not represent population specific selection events *per se*, and its length is not an indication of selection events in the Danish. This indicates that the ability of PBS to truly distinguish population specific allele frequency changes is dependent on the configuration of populations included in its computation. To show this is true, we plot the rank correlations of the three different PBS statistics possible for PopA. PBS_ABC_ and PBS_ABD_ are highly correlated (*spearman’s rho* = 0.82, Figure S11b) but both are poorly correlated with PBS_ACD_, which is a compound branch length in our model (*spearman’s rho* = 0.46 and 0.47; PBS_ACD_ vs. PBS_ABC_ and PBS_ABD_, Figure S11b).

That PBS_ABC_ and PBS_ABD_ are not perfectly correlated indicates that each contains independent information in delimiting the branch length of PopA, and illustrates the motivation in producing a statistic that draws upon the full four population F_ST_ matrix.

In deriving this statistic, we note that PBS is just a simple algebraic function of the matrix of pairwise F_ST_ values. To find PBS_ABC_, for example: PBS_ABC_ = (distanceAB + distanceAC – distanceBC)/2. An alternative method for finding distances in a phylogeny is the Neighbor-Joining algorithm (NJ) [3]. Without giving the full details, NJ proceeds by calculating a *Q* matrix from the input distance matrix, creating a node by grouping the two taxa with the smallest *Q*, and re-calculating distances with respect to the new node. In this sense, branch lengths are a by-product of the NJ procedure, but nonetheless, by recording these branch lengths for each SNP NJ tree across the genome, we can generate a distribution of branch lengths analogous to PBS. For this reason, we name this proposed statistic PBSnj. While the details and actual distances calculated differ, PBS and PBSnj both define a distance for each branch in a tree, and the correlation between three-population PBS and PBSnj branch lengths suggests that these two methods are near identical in their results (spearman’s *rho* = 0.995).

Extending PBS to more than three populations require fixing a topology. In the four population case, branch length A could be calculated as: PBS_ABCD_ = (PBS_ABC_ + PBS_ABD_) / 2, but this assumes that the tree at each site follows the species tree ((A,B), (C,D)). It also “hides” the presence of an internal branch implicit in a bifurcating four taxa tree. While more complicated sets of algebraic functions could be combined to solve this or other conundrums, it is enough to point out that nj does not assume a topology (it is after all a topology finder) and that its algebraic rules are consistent no matter the number of taxa, the only change being the number of repetitions of the algorithm. Thus, we conclude that PBSnj is the more natural method to use. Lastly, while we have not considered it here, in theory PBSnj is extendable to any number of taxa.

We also do not consider the internal branch as in this investigation we are only interested in the selection pressures that differentiate extant populations of chimpanzees. Furthermore, interpretation of the direction of the internal branch in the four taxa case relies again on assuming that the derived allele is the target of selection.

The schematic for calculating PBSnj is as follows:

1. for each site, calculate the full F_ST_ matrix.
2. apply the Neighbor-Joining algorithm on the F_ST_ matrix, i.e. generate a nj-tree.
3. for each site, record the branch length for each taxa in the nj-tree.

Following the original description of PBS [1], we transform the F_ST_ values into units of drift time: -ln(1-F_ST_). As this is undefined for F_ST_ == 1, we substitute F_ST_ == 1 for the next lowest possible pairwise F_ST_ value. So that branch lengths exhibit the same range, following [4] we standardised branch lengths by the total length of the tree, e.g. PBSnj_A_scaled_ = PBSnj_A_ / (1 + PBSnj_A_+ PBSnj_B_ + PBSnj_C_ + PBSnj_D_ + PBSnj_INTERNAL_). Lastly to perform genic enrichment tests analogous to derived allele frequency difference we re-scale so that values are within the range 0-1. This implies values of PBSnj >= 0.8 (which we use as our cut-off or PBSnj genic tail bin) are those equal to 80% or more of the max possible values of PBSnj, are not a quantile cut-off and can therefore contain a differing number of sites per taxa.

As a simple illustration of the effectiveness of PBSnj to identify population specific changes in allele frequency, we asked how well the statistics identify Pop A specific allele frequency change. We plot the derived allele frequency in each of Pops A-D, for those sites for which PBSnj_A_scaled_ >= 0.8 (Figure S11c). As a comparison, we do the same for PBS_ABC_, PBS_ABD_ and PBS_ACD_. PBSnj_A_ clearly delineates those sites specifically differentiated in Pop A. PBS_ACD_ is the worst test statistic, as Pop B allele frequencies are nearly uniformly distributed in the range of 0-1 despite these sites being identified as pop A outliers. PBS_ABC_ and PBS_ABD_ offer a substantial improvement, but note there is a tendency for a more uniform distribution of allele frequencies in the population not included in the calculation of PBS_ABC_ and PBS_ABD_ (D and C respectively). Note too, the point masses near 0 for Pop A, and near 1 for Pops B-D in PBSnj_A_scaled_ which represent those sites where PopA has a very low derived allele frequency i.e. are ancestral allele outliers.

1. Yi X, Liang Y, Huerta-Sanchez E, Jin X, Cuo ZXP, Pool JE, et al. Sequencing of 50 human exomes reveals adaptation to high altitude. Science. 2010;329(5987):75-8. doi: 10.1126/science.1190371.

2. Ewing G, Hermisson J. MSMS: a coalescent simulation program including recombination, demographic structure and selection at a single locus. Bioinformatics. 2010;26(16):2064-5. Epub 2010/07/02. doi: 10.1093/bioinformatics/btq322. PubMed PMID: 20591904; PubMed Central PMCID: PMCPMC2916717.

3. Saitou N, Nei M. The neighbor-joining method: a new method for reconstructing phylogenetic trees. Mol Biol Evol. 1987;4(4):406-25. Epub 1987/07/01. doi: 10.1093/oxfordjournals.molbev.a040454. PubMed PMID: 3447015.

4. Malaspinas AS, Westaway MC, Muller C, Sousa VC, Lao O, Alves I, et al. A genomic history of Aboriginal Australia. Nature. 2016;538(7624):207-14. Epub 2016/09/23. doi: 10.1038/nature18299. PubMed PMID: 27654914.
