# Supplementary material for: The impact of genetic adaptation on chimpanzee subspecies differentiation
Source: PLoS Genet. 2019 Nov 25;15(11):e1008485. doi: 10.1371/journal.pgen.1008485 (PMC6901233; doi:10.1371/journal.pgen.1008485)
Supplement: S5 Appendix — (DOCX) [file pgen.1008485.s005.docx]

# S5 Appendix.

## The relationship between divergence times and N_e_ and the effects of BGS.

Demography varies greatly amongst the chimpanzee sub-species, with a wide range of pairwise divergence times and effective population sizes *N_e_* (see Main Figure 1). This means that the total genetic drift between, for example, Western and Central chimpanzees is much greater than that between Central and Eastern chimpanzees. It is unknown how these differences in drift times effects genic enrichments in bins of either the signed differences in derived allele frequency (*δ*) or PBSnj. To explore this, we again used a simple four population model (described S4 Appendix). To model the effect of background selection (BGS) we can scale *N_e_* by a value B, such that *B*= 0.9, for example, represents BGS that reduces linked neutral diversity by 10%. Genic regions were simulated using B = 0.9. We also allowed either the basal split time, or the split time of pop1 and pop2 to increase, therefore widening the range of divergence times.

For each scenario, we simulated 50 chromosomes per deme for a 2kb locus, for 2 million replicates, using a mutation rate of 1.2 x 10^-8^ and recombination rate of 0.96 x 10^-8^.

**The effect of divergence time and N_e_ on δ tail bin SNP n.**

Increasing the divergence time increases the number of SNPs in both genic and non-genic tail bins, as is expected due to a greater variance in allele frequency due to genetic drift. While intuitive, it is important to demonstrate as it shows that the number of SNPs in tail bin is not itself an indication of the statistical support for selection. Increasing divergence time also reduces the genic:non-genic SNP n ratio: from ~1.5 at time = 0.1 down to ~1.08 at time = 0.4.

Changes in *N_e_* also greatly affect the number of *δ* tail bin SNPs, see S24 Table. We varied the simulated PopB:PopA ratio to be either 0.9, 0.5 or 0.1. On this time scale, an *N_e_* ratio of 0.9 has a modest impact on the number of Pop2 tail bin SNPs. However, ratios of 0.5 and 0.1 result in a dramatic increase in both Pop2 genic and non-genic tail bin SNPs. Of course, this mirrors the result of increasing divergence time – for the same evolutionary time, lower *N_e_* results in greater drift. What is also apparent is that that the lowered Pop2 *N_e_* also results in an increase in the Pop1 *δ* tail bin counts. When Pop2 *N_e_* = 0.1, the ratio of genic to non-genic *δ* tail SNPs is ~ 1 for both populations. We posit that these two factors – increased divergence and lower effective population size – explain the lower genic enrichments seen for *δ* calculated with western chimpanzees. A secondary point is the implication that the genic enrichment produced by a given strength of BGS decreases with drift time.

**The effect of divergence time and N_e_ on PBSnj tail bin genic enrichment**

Divergence time and *N_e_* impact *δ* tail genic enrichment of both populations. This is because it conflates allele frequency change occurring in two populations. In contrast, PBSnj is able to determine the allele frequency change that occurs specifically in one branch of a phylogeny.

To show the effect of *N_e_* on the PBSnj tail genic enrichment, we plot the genic enrichment assuming BGS of B =0.9 but varying the *N_e_* of Pop2 (Figure S11, S25 Table). Given a relative *N_e_* = 1, the genic enrichment in the PBSnj tail bin = 1.20, and there is no effect in reducing the *N_e_* to 0.9 (Figure S11). Below *N_e_* = 0.9, the genic enrichment drops precipitously to 1.16 for *N_e_* = 0.5 and 1.06 for *N_e_* = 0.1. We suggest that this shows that BGS has a greater impact when divergence times are shorter and *N_e_* relatively large, that is when most of the variation between lineages is still segregating. Longer divergence times and lower *N_e_* results in a greater number of fixed differences between lineages, and BGS does not impact the divergence in genic regions to the extent that it reduces diversity and distorts the SFS of segregating variation.

This result is the motivation for comparing only central and eastern chimpanzee PBSnj tail genic enrichments. Not only is their pairwise divergence time the lowest amongst the chimpanzees, but given their relative *N_e_*_,_ we would not expect *N_e_* to be the reason that eastern chimpanzees exhibit a greater PBSnj tail genic enrichment. Indeed, simulations recapitulating the demographic history of chimpanzees suggest that BGS produces equal genic enrichments for eastern and central chimpanzees. As well as expecting a similar level of drift in each of their branches, given a constant rate of adaptive evolution, we would also expect a similar number of adaptive events to contribute to the genic enrichment.
