# Supplementary material for: The impact of genetic adaptation on chimpanzee subspecies differentiation
Source: PLoS Genet. 2019 Nov 25;15(11):e1008485. doi: 10.1371/journal.pgen.1008485 (PMC6901233; doi:10.1371/journal.pgen.1008485)
Supplement: S6 Appendix — (DOCX) [file pgen.1008485.s006.docx]

# S6 Appendix

## Estimating the strength of background selection required to explain PBSnj tail genic enrichments in chimpanzees.

We also determined how background selection can affect the PBSnj statistic amongst chimpanzees and found that they each have a unique value of B which best explains their PBSnj tail bin genic enrichment. We explain this by positive selection differentially influencing the tail of each species, as we do not expect [1] or observe differences in the effects of background selection across species. Simulating BGS using variable B, both the Eastern and Nigeria-Cameroon PBSnj tail genic enrichments can be explained with similar strengths of BGS. The 90% CI of the observed genic enrichment is achievable with BGS in the range of 0.9-0.92 for eastern and 0.89-0.91 for Nigeria-Cameroon (S6 Table). However, using a single average B value, only eastern chimpanzees require a B stronger than 0.888 to achieve the observed PBSnj tail genic enrichment (S6 Table).

Of critical importance for interpreting the greater PBSnj tail genic enrichment for eastern compared to that for central chimpanzees is the observation that across all values of B tested, simulated genic enrichments are approximately identical for these two subspecies or greatest for central chimpanzees (Figure 4). Thus, demography and BGS should not produce the observed pattern of greater eastern genic enrichment. Results from our generalised four population model also indicate that the relatively small difference between eastern and central *N_e_* are also not a likely explanation. In fact, such differences should result in a higher enrichment for central chimpanzees who have the larger *N_e_*. We again posit that this is evidence for a greater rate of adaptive events along the eastern branch than that for the central branch.

In contrast, for any given strength of BGS, the simulated eastern and central genic enrichments are always greater than those of western and Nigeria-Cameroon chimpanzees (S11 Figure). Our explanation for this is as follows: most tail SNPs for Nigeria-Cameroon, and especially for western chimpanzees, are actually fixed differences to all other chimpanzees. On the other hand, most eastern and central PBSnj tail SNPs are polymorphisms shared between these two sub-species. In addition, results from our general four population model, indicate that by increasing the lineage specific drift by increasing divergence time and/or decreasing *N_e_*, the genic enrichment caused by BGS decreases. Again, we suggest that this indicates that BGS is more important for polymorphism than divergence. Finally, we conclude that only the eastern vs. central PBSnj tail bin comparison is informative in judging the significance of the eastern PBSnj tail genic enrichment or the likelihood that this can be explained by BGS.

1. Nam K, Munch K, Mailund T, Nater A, Greminger MP, Krützen M, et al. Evidence that the rate of strong selective sweeps increases with population size in the great apes. Proceedings of the National Academy of Sciences. 2017;114(7):1613-8. doi: 10.1073/pnas.1605660114.
