# Supplementary material for: The impact of genetic adaptation on chimpanzee subspecies differentiation
Source: PLoS Genet. 2019 Nov 25;15(11):e1008485. doi: 10.1371/journal.pgen.1008485 (PMC6901233; doi:10.1371/journal.pgen.1008485)
Supplement: S7 Appendix — (DOCX) [file pgen.1008485.s007.docx]

# S7 Appendix.

**Demography and the evidence of positive selection in central chimpanzees**

We use this section to also discuss the factors that could blunt the evidence for positive selection in central chimpanzees. Population structure within the sampled central chimpanzees could reduce the apparent number of highly differentiated alleles. Fixed beneficial alleles in two divergent central chimpanzee populations would, for example, look to be segregating at intermediate frequencies if these were both sampled equally. Population structure within chimpanzee subspecies has been extensively analysed by both [1] and [2]. de Manuel et. al. (2016) present the results of numerous analyses within their supplementary material, including results from sNMF [3], fineSTRUCTURE [4], and ADMIXTURE [5] (respectively figures S11, S13 and S14 in de Manuel et. al. (2016)). For each of these analyses, there is less structuring of the sampled central chimpanzees compared to the sampled eastern chimpanzees, which in contrast often appear as a cline of variation from Tanzania and the south of the Democratic Republic of the Congo (DRC) through to Uganda and northern DRC. This suggests that unaccounted-for population structure is not a reason for weaker genic enrichment of differentiated alleles in central chimpanzees.

Another possible blunting mechanism is gene flow. When simulating neutral evolution and BGS, we used coalescent simulations using demographic parameters previously described in de Manuel et. al. (2016). This model includes inferred gene flow amongst Pan lineages, including that of the bonobo – central chimpanzee introgression. However, what these simulations do not address is the possibility that alleles selected in central chimpanzees were constantly stopped from reaching fixation due to the introduction of bonobo alleles until cessation of this gene flow ~40 kya. We note first, that gene flow is a general barrier to local adaptation, and that the rate of migration and strength of selection are the two key parameters determining the likelihood of reaching fixation. If gene flow into central chimpanzees was too great or selection too weak, then this could reduce the genic enrichment in population specific, highly differentiated alleles – but this would reflect the biological reality of reduced local adaptation in this subspecies. Secondly, we highlight that while bonobo introgression into central chimpanzees did occur, the scale of this gene flow is dwarfed by the ongoing, and near symmetrical, gene flow between central and eastern chimpanzees (migration into central chimpanzees from bonobo was ~ 1.6% of the ongoing rate from eastern chimpanzees, and ~ 1.9% of the ongoing rate of migration from central into eastern chimpanzees). These rates of migration would pose a greater barrier to adaptive population differentiation, and the signal in eastern chimpanzees is identified despite this.

1. Prado-Martinez J, Sudmant PH, Kidd JM, Li H, Kelley JL, Lorente-Galdos B, et al. Great ape genetic diversity and population history. Nature. 2013;499(7459):471-5. doi: 10.1038/nature12228.

2. De Manuel M, Kuhlwilm M, Frandsen P, Sousa VC, Desai T, Prado-Martinez J, et al. Chimpanzee genomic diversity reveals ancient admixture with bonobos. Science. 2016;354(6311):477-81. doi: 10.1126/science.aag2602.

3. Frichot E, Mathieu F, Trouillon T, Bouchard G, Francois O. Fast and efficient estimation of individual ancestry coefficients. Genetics. 2014;196(4):973-83. Epub 2014/02/06. doi: 10.1534/genetics.113.160572. PubMed PMID: 24496008; PubMed Central PMCID: PMCPMC3982712.

4. Lawson DJ, Hellenthal G, Myers S, Falush D. Inference of population structure using dense haplotype data. PLoS Genet. 2012;8(1):e1002453. Epub 2012/02/01. doi: 10.1371/journal.pgen.1002453. PubMed PMID: 22291602; PubMed Central PMCID: PMCPMC3266881.

5. Alexander DH, Novembre J, Lange K. Fast model-based estimation of ancestry in unrelated individuals. Genome Res. 2009;19(9):1655-64. Epub 2009/08/04. doi: 10.1101/gr.094052.109. PubMed PMID: 19648217; PubMed Central PMCID: PMCPMC2752134.
