# Supplementary figures and images for: The impact of genetic adaptation on chimpanzee subspecies differentiation
Source: PLoS Genet. 2019 Nov 25;15(11):e1008485. doi: 10.1371/journal.pgen.1008485 (PMC6901233; doi:10.1371/journal.pgen.1008485)

a

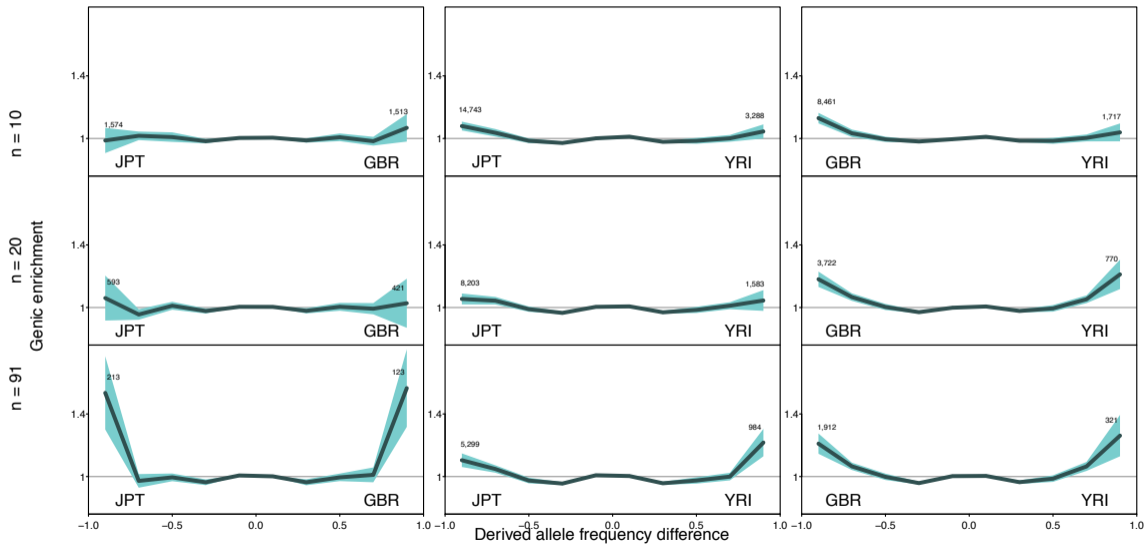

b

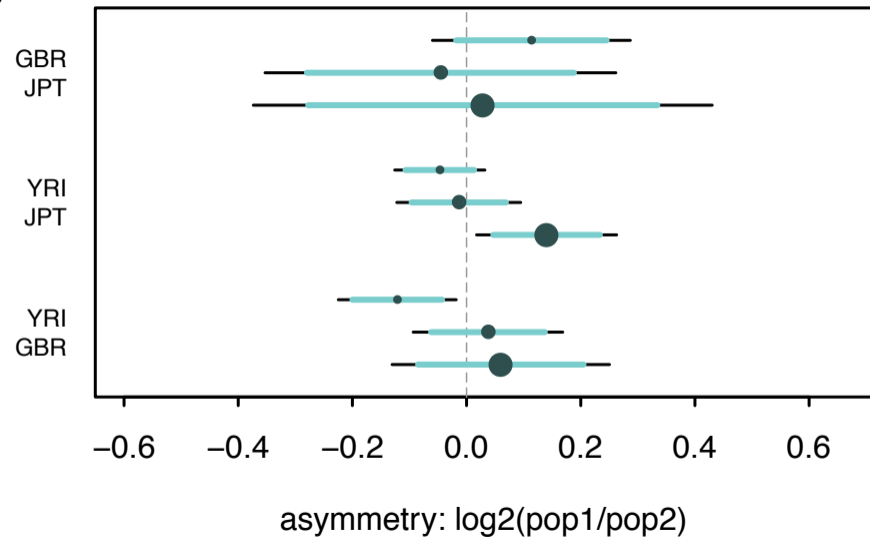

Supplement: S1 Fig — a, X-axis: δ is computed as the difference in derived allele frequency, for each pair of populations. Tail bins (the last bin in either end of δ) contain those SNPs with the largest allele frequency differences. Numbers are of the genic SNPs in each tail bin. Y-axis: genic enrichment in each δ bin, computed as described in Methods. Shading represents the 95% CI (i.e. alpha = 0.05 for a two-tailed test) estimated by 200kb weighted block jackknife, b, The asymmetry of the genic enrichments in the δ tails is measured by taking their log2 ratio, thus 0 indicates a symmetric enrichment (equal enrichment in both δ tails). Dot = observed asymmetry, with size indicating the relative sample size (10, 20, 91 individuals). Horizontal lines represent confidence intervals estimated by 200kb weighted block jackknife (light = 95%, black = 99%, i.e. alpha = 0.05 or 0.01 for a two-tailed test). (PDF) [file pgen.1008485.s008.pdf]

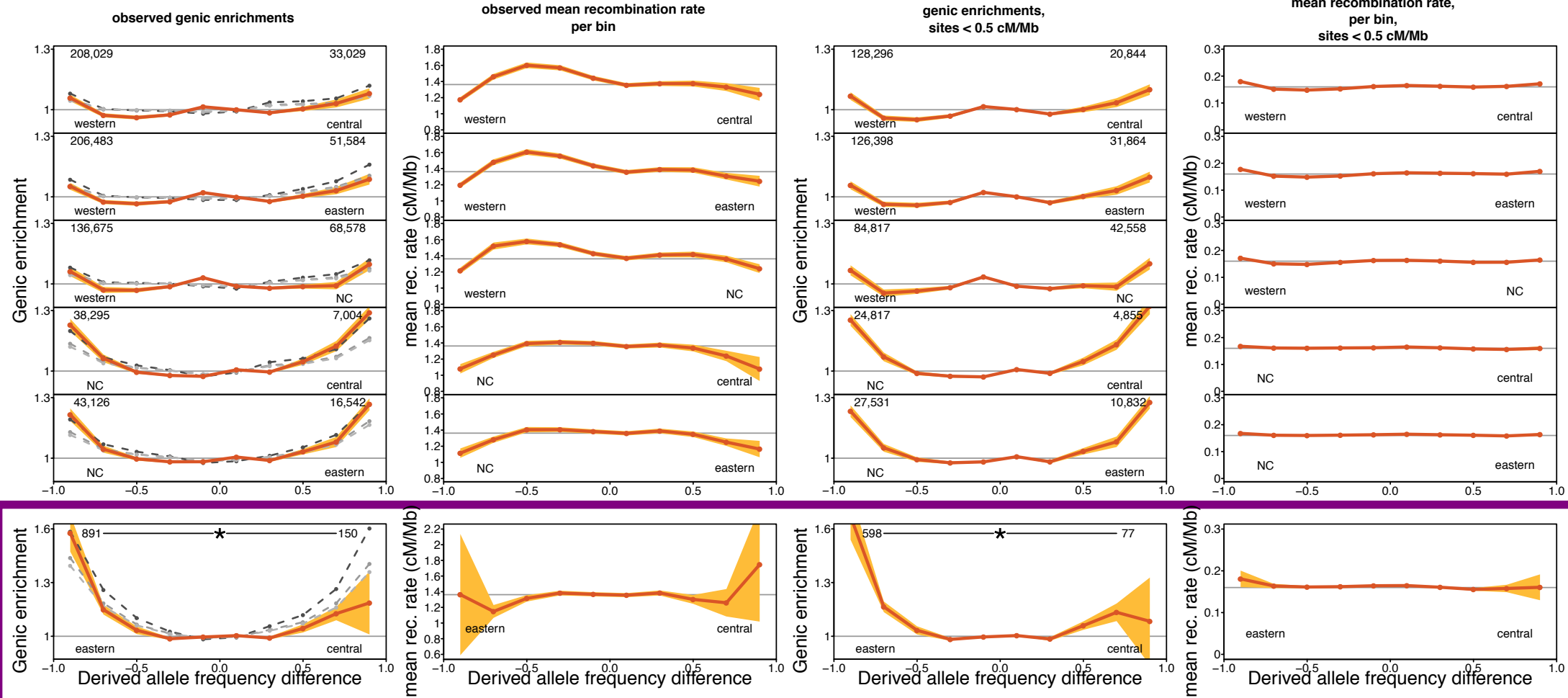

Supplement: S2 Fig — X axes: Binned δ, the difference in derived allele frequency, for each pair of populations. Y-axes: columns one and three: genic enrichment in each δ bin; columns two and four: mean genic recombination rate for each δ bin. Columns one and two: observed data. Columns three and four: analysis restricted to sites with recombination rate < 0.5 cM/Mb. Shading represents the 95% CI (i.e. alpha = 0.05 for a two-tailed test) estimated by 200kb weighted block jackknife. Light grey horizontal line represents: columns one and three expected genic enrichment; columns two and four, mean genic recombination rate. (PDF) [file pgen.1008485.s009.pdf]

**A**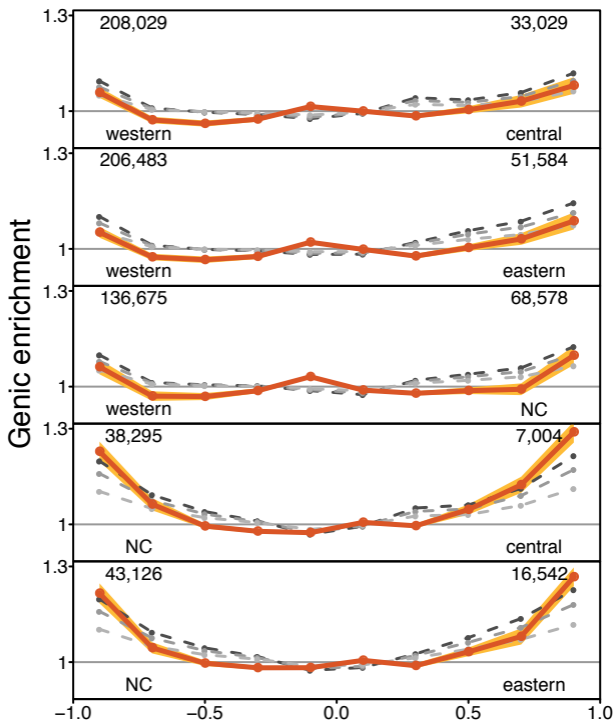**B**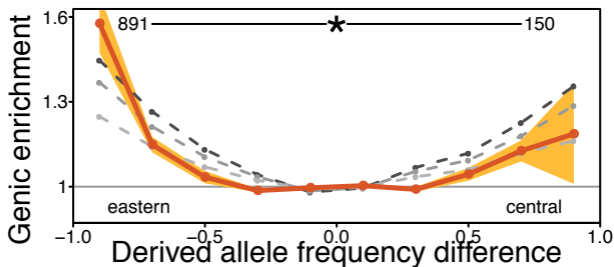

Supplement: S3 Fig — A, X-axis: δ is computed as the difference in derived allele frequency, for each pair of chimpanzee subspecies. Tail bins (the last bin in either end of δ) contain those SNPs with the largest allele frequency differences. Numbers are of the genic SNPs in each tail bin. Y-axis: genic enrichment in each δ bin (Methods). B, Genic enrichment eastern and central chimpanzee δ, plotted separately due to a different Y-axis limit. NC = Nigeria-Cameroon. The asterisk shows significance of the asymmetry in the genic enrichment (* = 0.01). Shading represents the 95% CI (i.e. alpha = 0.05 for a two-tailed test) estimated by 200kb weighted block jackknife. Grey dashed lines represent simulations under increasing levels of background selection that best match different aspects of the data: lightest to darkest shades: B = 0.925 (excluding δ tail bins), 0.888 (all δ bins), and 0.863 (only δ tail bins). (PDF) [file pgen.1008485.s010.pdf]

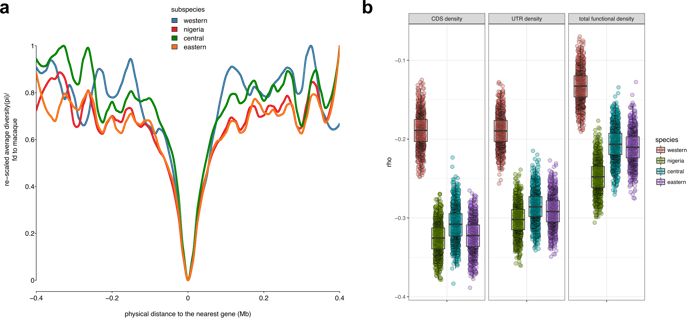

Supplement: S4 Fig — a, Diversity levels at neutral sites as a function of the distance to the nearest gene. We calculated scale diversity (pi / divergence to macaque) in bins of distance to genic regions. We then rescaled scaled diversity for each subspecies so that the diversity was in the range 0–1. b, To further explore the effects of BGS on chimpanzee genomes we checked the correlation of density of functional sites with neutral diversity (pi). We used windows 500kb spaced at least 1MB apart in the genome. Here, rho is the spearman rank partial correlation between windowed diversity and density of functional sites per window, controlling for recombination rate (the average rate per window). Each dot represents a bootstrap replicate (random sample of 500 kb windows). We calculated the partial rho for each bootstrap. Box plots show the median and interquartile ranges of the bootstrap replicates. (PNG) [file pgen.1008485.s011.png]

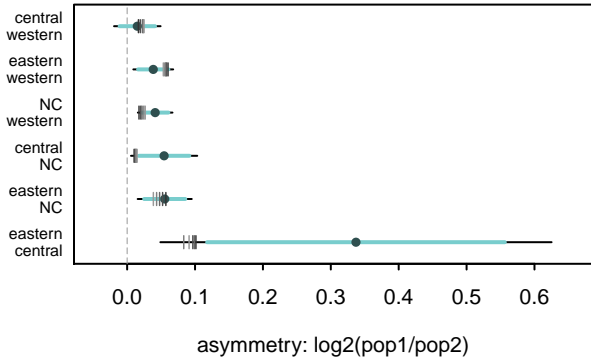

Supplement: S5 Fig — The asymmetry of the genic enrichments in the δ tails is measured by taking their log2 ratio, thus 0 indicates a symmetric enrichment (equal enrichment in both δ tails). We created coalescent simulations in which the strength of BGS was greater in eastern chimpanzees than other subspecies. For eastern chimpanzees we chose a fixed B = 0.825, as this B provided the best fit the eastern δ tail genic enrichment. All other subspecies had the same B, in the range of 0.900–0.850. A larger difference in B between subspecies results in a slight increase in asymmetry, but none of the simulated differences in BGS result in the observed asymmetry. Point = observed asymmetry. Horizontal lines represent confidence intervals estimated by 200kb weighted block jackknife (light = 95%, black = 99%, i.e. alpha = 0.05 or 0.01 for a two-tailed test). Grey vertical marks represent the δ tail asymmetry in simulations, under increasing levels of difference in background selection between eastern and other chimpanzees: lightest to darkest shades: All Beastern = 0.825; Bothers = 0.850, 0.863, 0.850, 0.888, 0.900. (PDF) [file pgen.1008485.s012.pdf]

**a**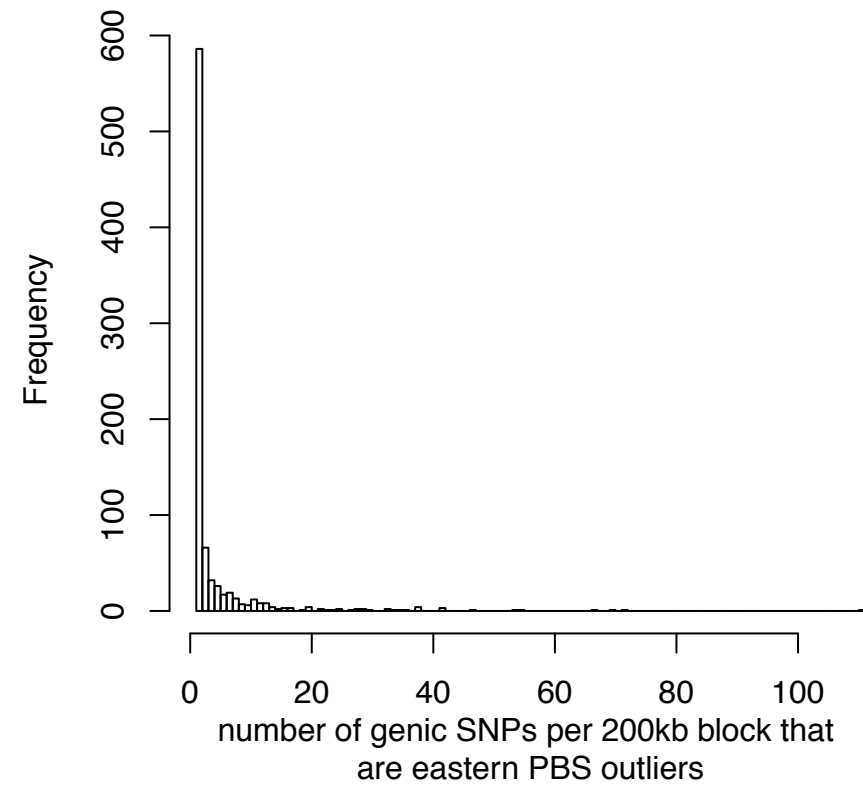**b**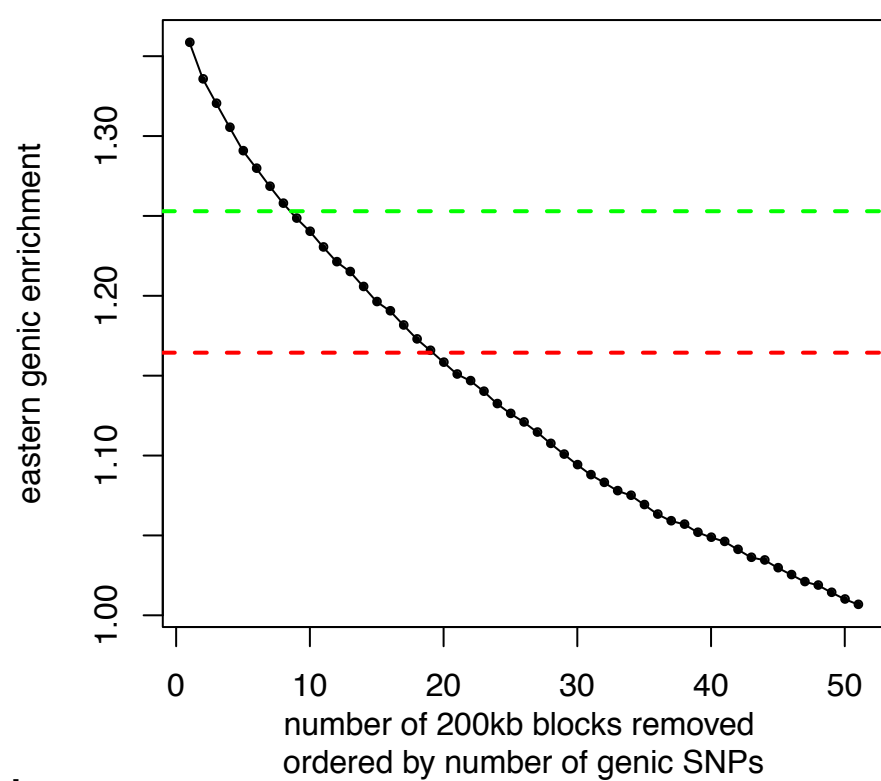**c**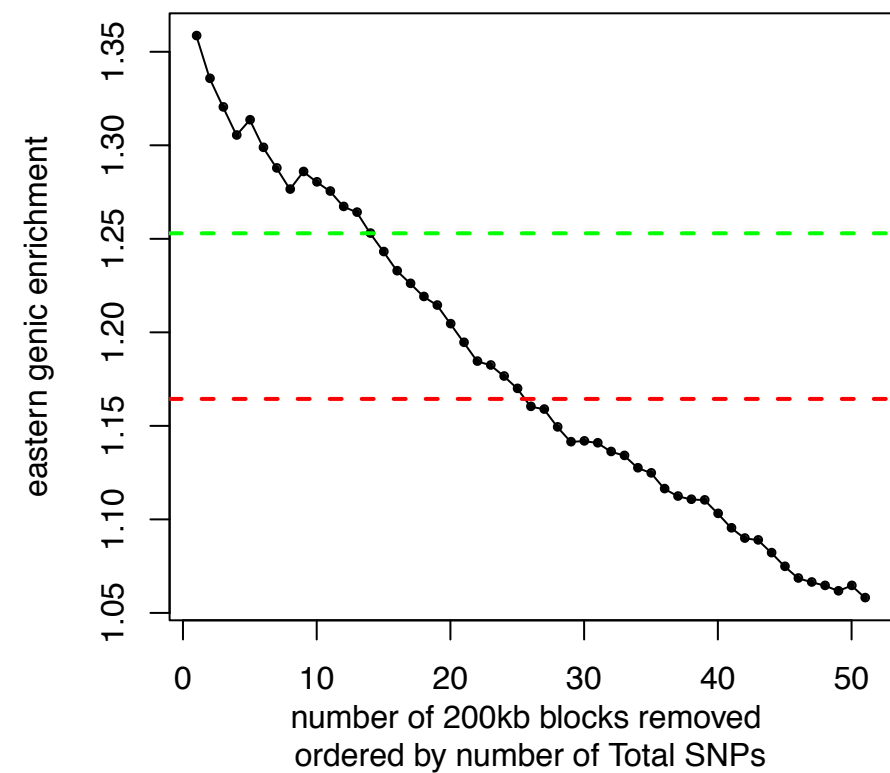**d**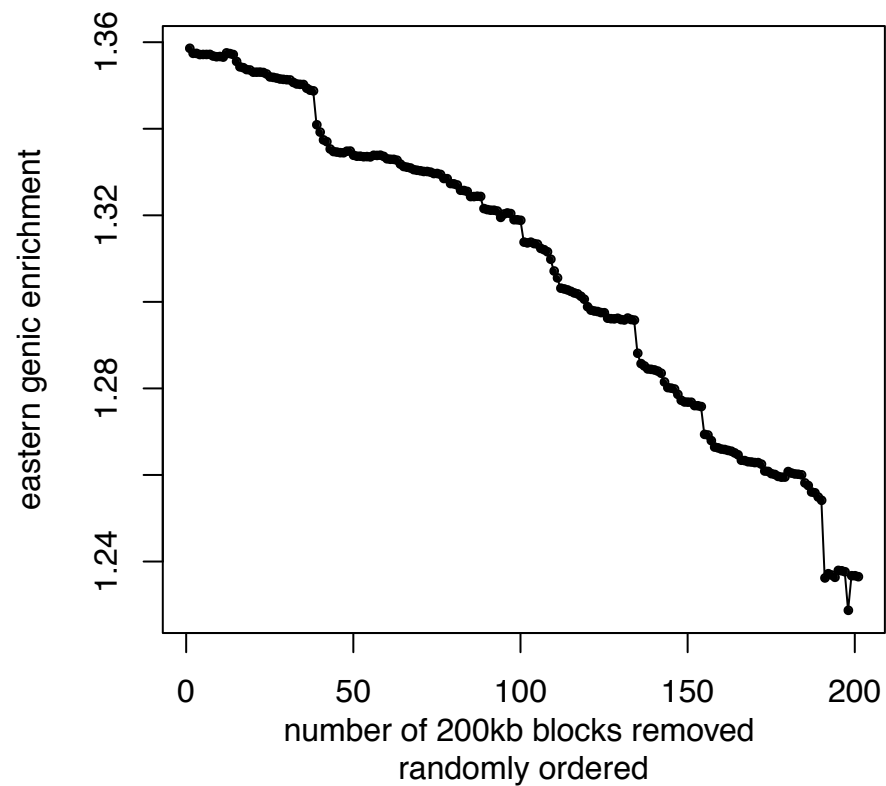

Supplement: S7 Fig — a, Most 200kb blocks contain few PBSnj eastern outlier SNPs, but there is an extended right hand tail. b, we ranked blocks by the number of PBSnj eastern tail SNPs, then iteratively removed outlier genic SNPs. This results in a monotonically decreasing genic enrichment, and the removal of eight blocks is required to reduce the genic enrichment of the PBSnj eastern tail to overlap the 95% CI of the PBSnj central tail, and 19 blocks to reduce it below the level of the point estimate of the central PBSnj tail. We could alternatively order the windows by the total number of outlier SNPs i.e. without regard to genic vs. non-genic. Doing so increases our estimated range of sweeps to 15–26. But we note that the genic enrichment does monotonically decrease with block removal (c). This is partly due to the arbitrary nature of the definition of genic, as it implies that there are some 200 kb blocks that have more non-genic than genic outlier SNPs contained within them, and this may very well change if the definition of genic was changed from transcription start and end sites +- 2kb. (d) Lastly, we randomly shuffled the removal order of the 200-kb blocks. We did so for 1000 random shuffles of the block order (A single random shuffle is shown). We find that the median number of blocks (i.e. sweeps) across random shuffles is 165 to match the upper 95% CI of the central chimpanzee estimate (middle 90% quantile range 114–221; min = 78, max = 278) increased to 273 to match the central chimpanzee point estimate of genic enrichment (middle 90% quantile range 214–329; min = 162, max = 381). Such a procedure is likely an overestimate, as most of the removal steps are those removing 1 to 9 genic outlier SNPs (panel a), resulting in minimal reduction of the genic enrichment. (PDF) [file pgen.1008485.s014.pdf]

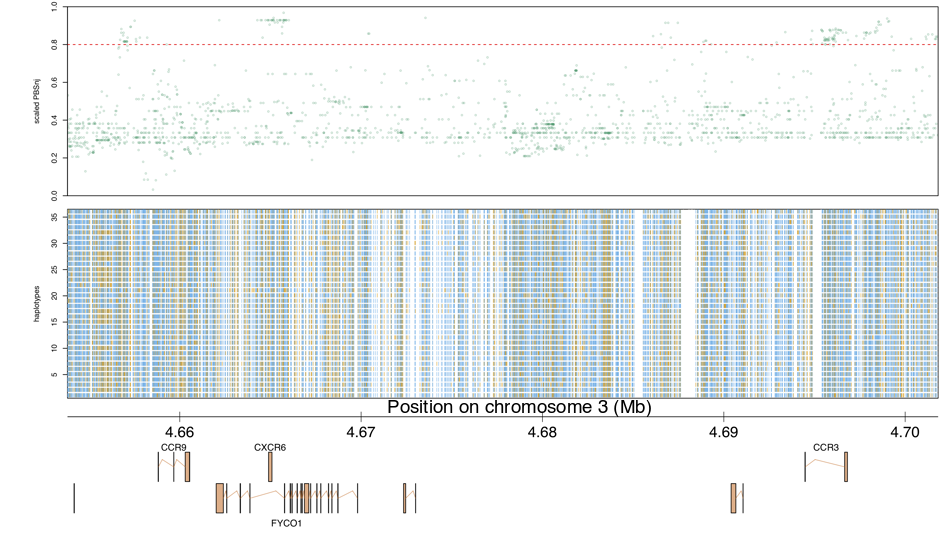

Supplement: S8 Fig — X axis: position along chromosome 3 (Mb). Plotted in the upper panel are the PBSnj central scores in the region encompassing CCR3, CCR9, and CXCR6. An independent cluster of high PBSnj scores is associated with each candidate gene. Each point represents one PBSnj score, colour has an alpha = 30% to reduce over plotting. Haplotypes are plotted in the central panel. Yellow ticks are derived alleles, blue are ancestral, while white is space so that each tick aligns with PBSnj scores. Inspection indicates that there is a degree of haplotype scrambling between each of the candidate genes. Lastly, we depict the genes in this region in the lower panel. (PNG) [file pgen.1008485.s015.png]

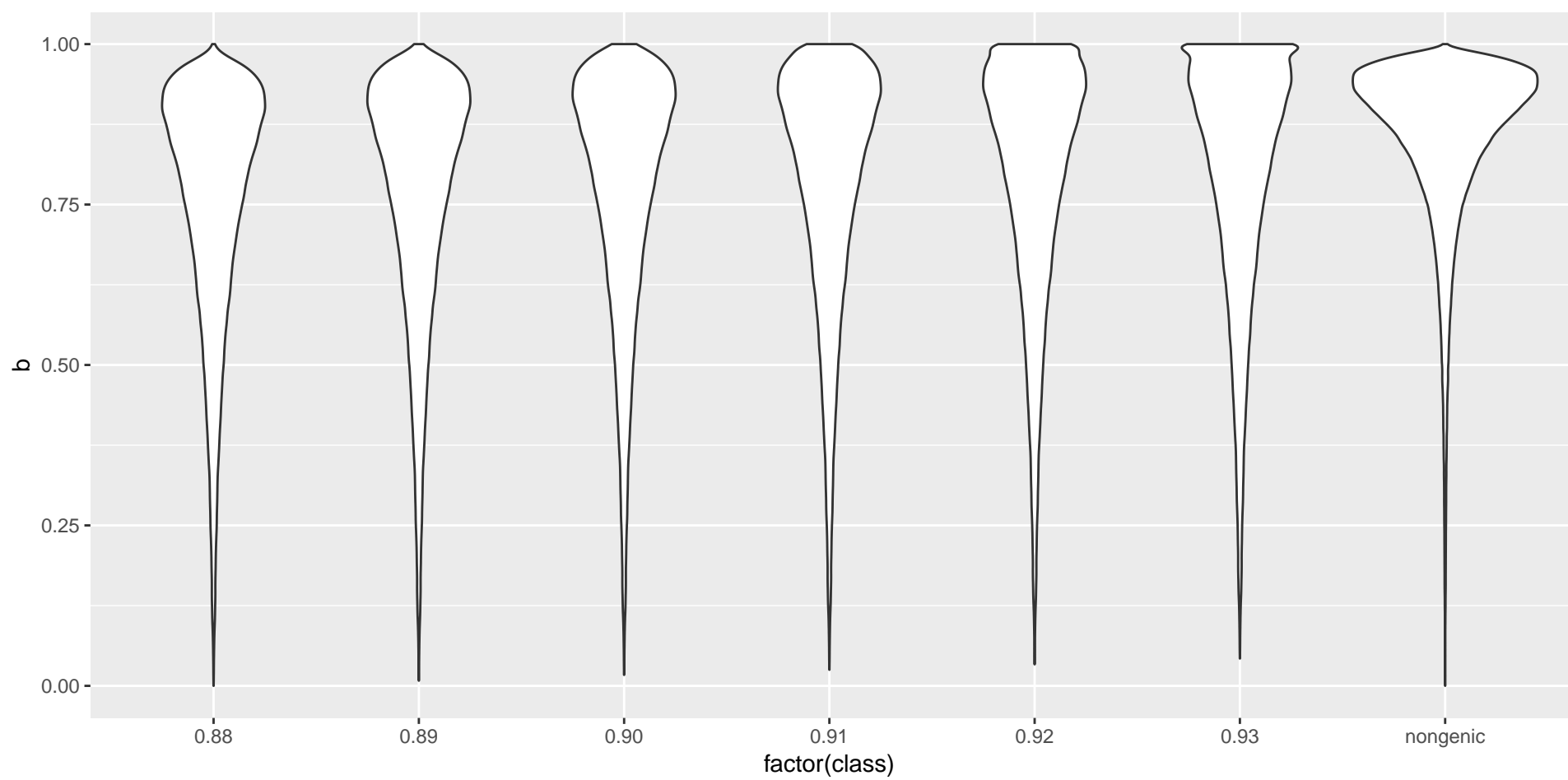

Supplement: S10 Fig — Violin plots are used to visualise the distribution of B values for non-genic and genic sites from McVicker, designated non-genic an “0.88” respectively. B = 0.88 is the average B ratio of genic / non-genic B values. We added fixed constants to all genic B values to modify the genome average B, in 0.01 steps from 0.99–0.93. (PDF) [file pgen.1008485.s017.pdf]

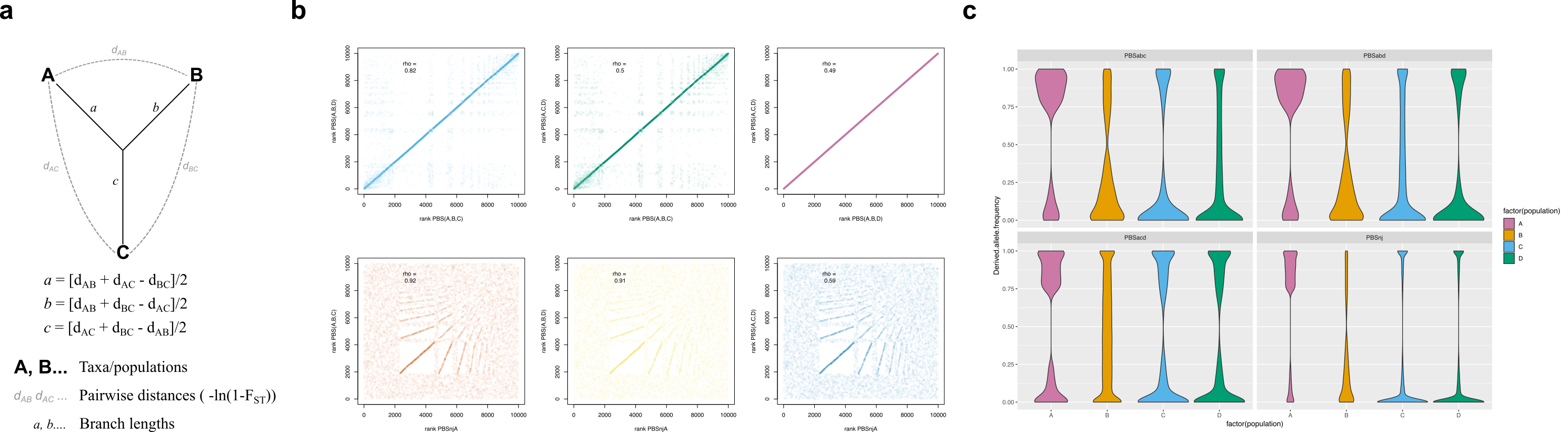

Supplement: S11 Fig — a, PBS is just a simple arithmetic function of pairwise FST values for a group of three taxa or populations. b, The configuration or choice of populations determines the information content of PBS. In each panel are the spearman’s rho correlations between different PBS configurations, and between PBS and our new statistic PBSnj for a simple four population model (described in Appendix 4). In each case Pop A is the focal population. PBSABC and PBSABD are highly correlated but not identical indicating that incorporating both Pops C and D would refine the identification of Pop A specific differentiated variants. PBSnjA, which utilises information from all four populations is more highly correlated with both PBSABC and PBSABD than they are with each other. Alpha = 10% for plotted points to reduce over saturation. c, For each statistic, we plot the site frequency spectrum (SFS) for each of the four populations for sites identified as outliers in Pop A. PBSnj clearly finds those sites differentiated in Pop A, and better than either PBSABC and PBSABD. In the standard PBS, the SFS in the species not included in the PBS configuration has a more uniform distribution, indicating that some sites identified as PBS frequency outliers in Pop A are not true population specific outliers. (PNG) [file pgen.1008485.s018.png]

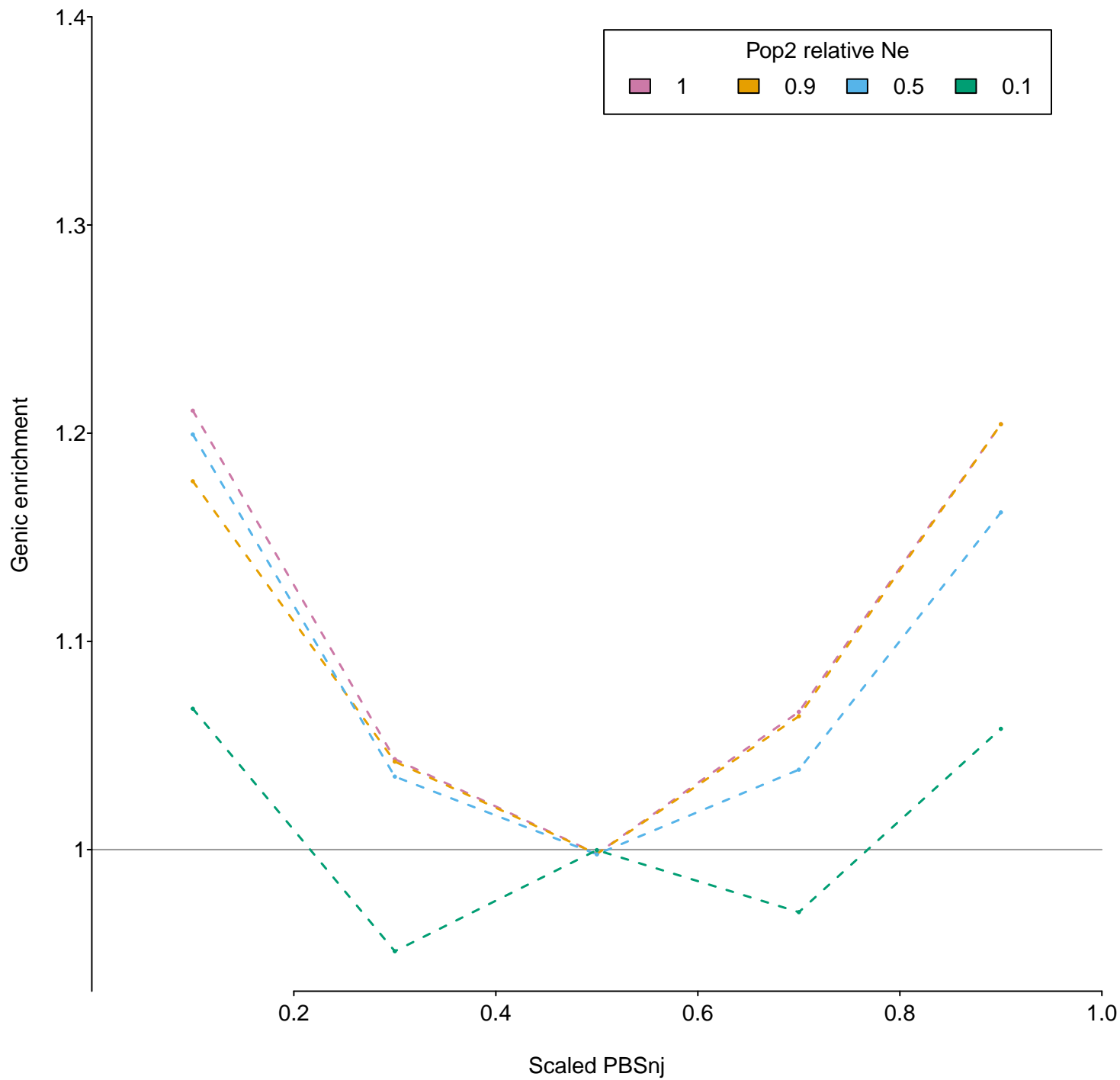

Supplement: S12 Fig — In a simple four population model, we modelled genic regions as those with a B = 0.9. In population 2, we simulated four effective population size ratios (1, 0.9, 0.5, 0.1). Ne ratios of 0.5 and 0.1 result in a reduced genic enrichment given the same strength of background selection. X-axes: PBS scaled to take values in the range 0–1, per subspecies. Y-axes: Genic enrichment. (PDF) [file pgen.1008485.s019.pdf]

**A**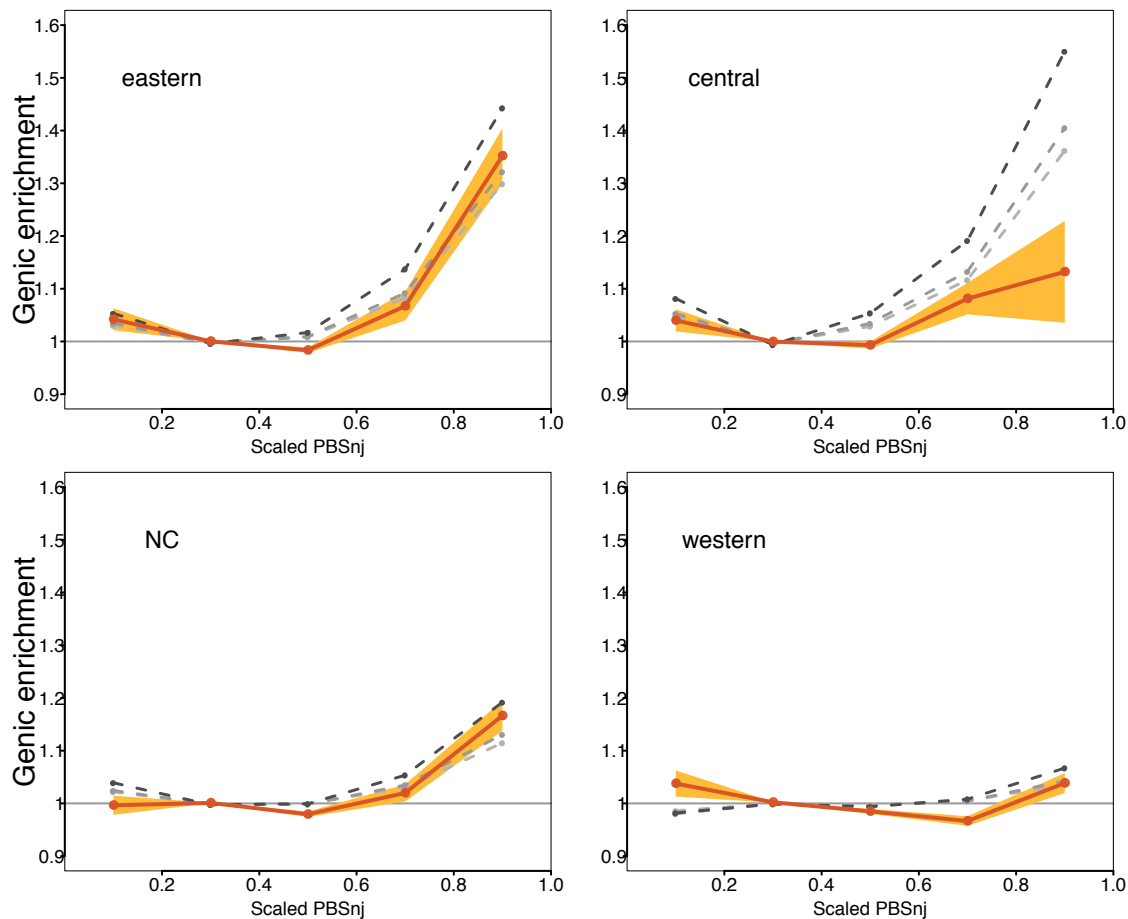**B**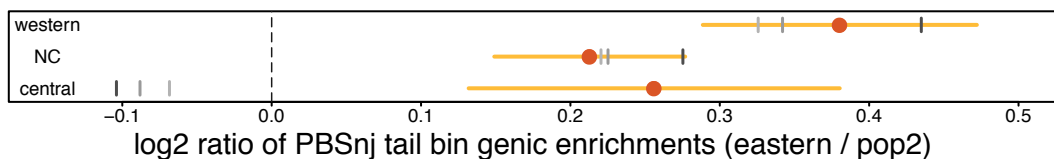

Supplement: S13 Fig — PBS scaled to take values in the range 0–1. Y-axes: Genic enrichment computed as described in Fig 2. Shading represents the 95% CI (i.e. alpha = 0.05 for a two-tailed test) estimated by 200kb weighted block jackknife. B: log2 ratio of the eastern versus western, Nigeria-Cameroon and central PBSnj tails (PBS > = 0.8) genic enrichment. A,B Grey dashed (A) or vertical (B) lines represent the PBSnj genic enrichment in simulations, under increasing levels of background selection that best match different aspects of δ, as described in Figs 2 and 3: lightest to darkest shades: B = 0.93 (excluding δ tail bins), 0.92 (all δ bins), and 0.88 (unmodified genic B values form McVicker). (PDF) [file pgen.1008485.s020.pdf]
