# Supplementary material for: The impact of genetic adaptation on chimpanzee subspecies differentiation
Source: PLoS Genet. 2019 Nov 25;15(11):e1008485. doi: 10.1371/journal.pgen.1008485 (PMC6901233; doi:10.1371/journal.pgen.1008485)
Supplement: S6 Fig — X axes: Binned PBSnj, for each subspecies. Y-axes: columns one and three: genic enrichment in each PBSnj bin; columns two and four: mean genic recombination rate for each PBSnj bin. Columns one and two: observed data. Columns three and four: analysis restricted to sites with recombination rate < 0.5 cM/Mb. Shading represents the 95% CI (i.e. alpha = 0.05 for a two-tailed test) estimated by 200kb weighted block jackknife. Light grey horizontal line represents: columns one and three expected genic enrichment; columns two and four, mean genic recombination rate. (PDF) [file pgen.1008485.s013.pdf]

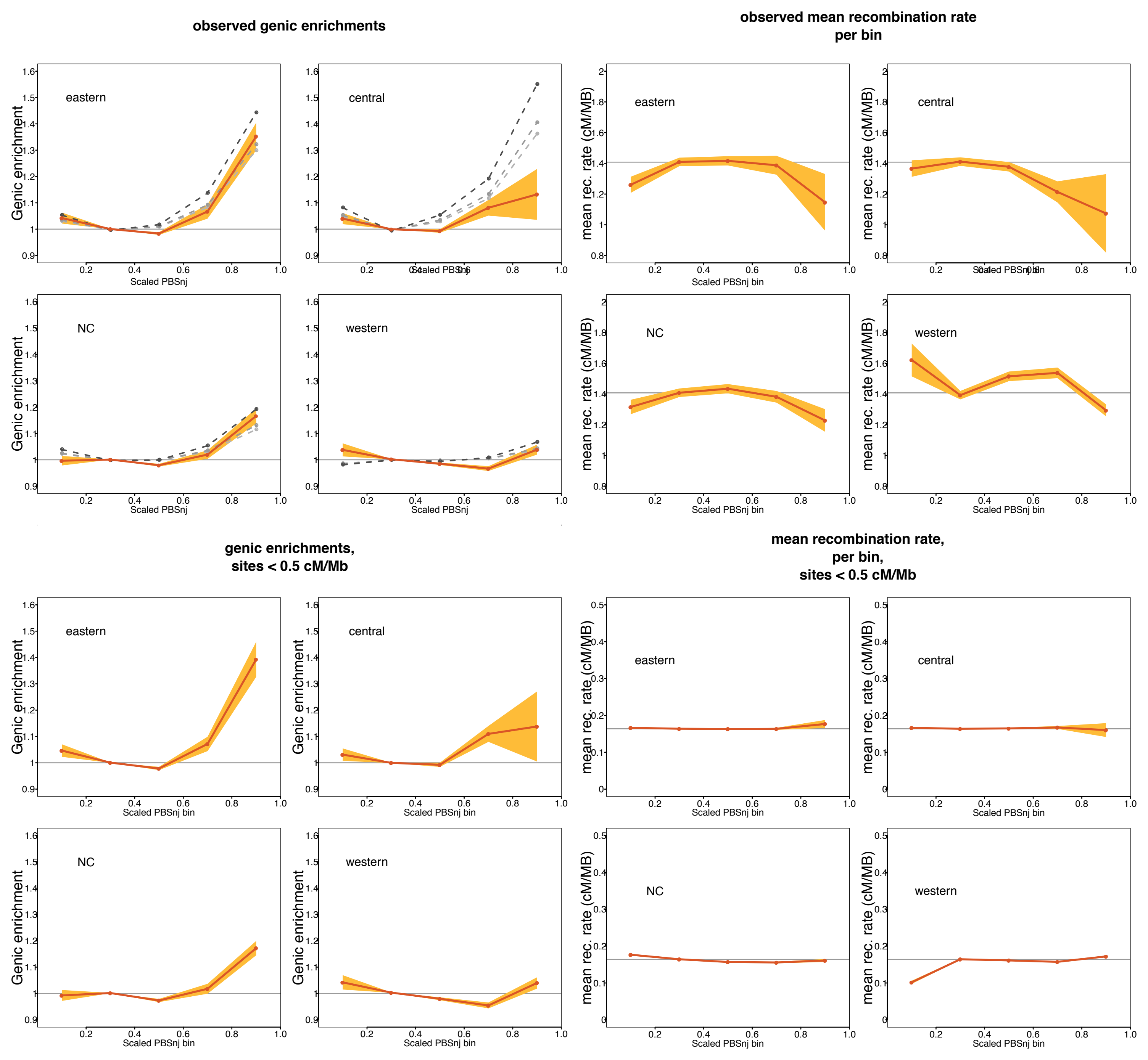

observed genic enrichments
observed mean recombination rate per bin
genic enrichments, sites < 0.5 cM/Mb
mean recombination rate, per bin, sites < 0.5 cM/Mb
eastern
central
NC
western
Genic enrichment
mean rec. rate (cM/MB)
Scaled PBSnj
Scaled PBSnj bin
