# Supplementary material for: The impact of genetic adaptation on chimpanzee subspecies differentiation
Source: PLoS Genet. 2019 Nov 25;15(11):e1008485. doi: 10.1371/journal.pgen.1008485 (PMC6901233; doi:10.1371/journal.pgen.1008485)
Supplement: S9 Fig — We plot the Site Frequency Spectrum (SFS) for each chimpanzee subspecies. X axes: derived allele count. Y axes: proportion. Black: observed. Green: simulated. Simulated counts come from 25 million 2kb loci simulated with msms, using the chimpanzee demography specified in Methods. (PDF) [file pgen.1008485.s016.pdf]

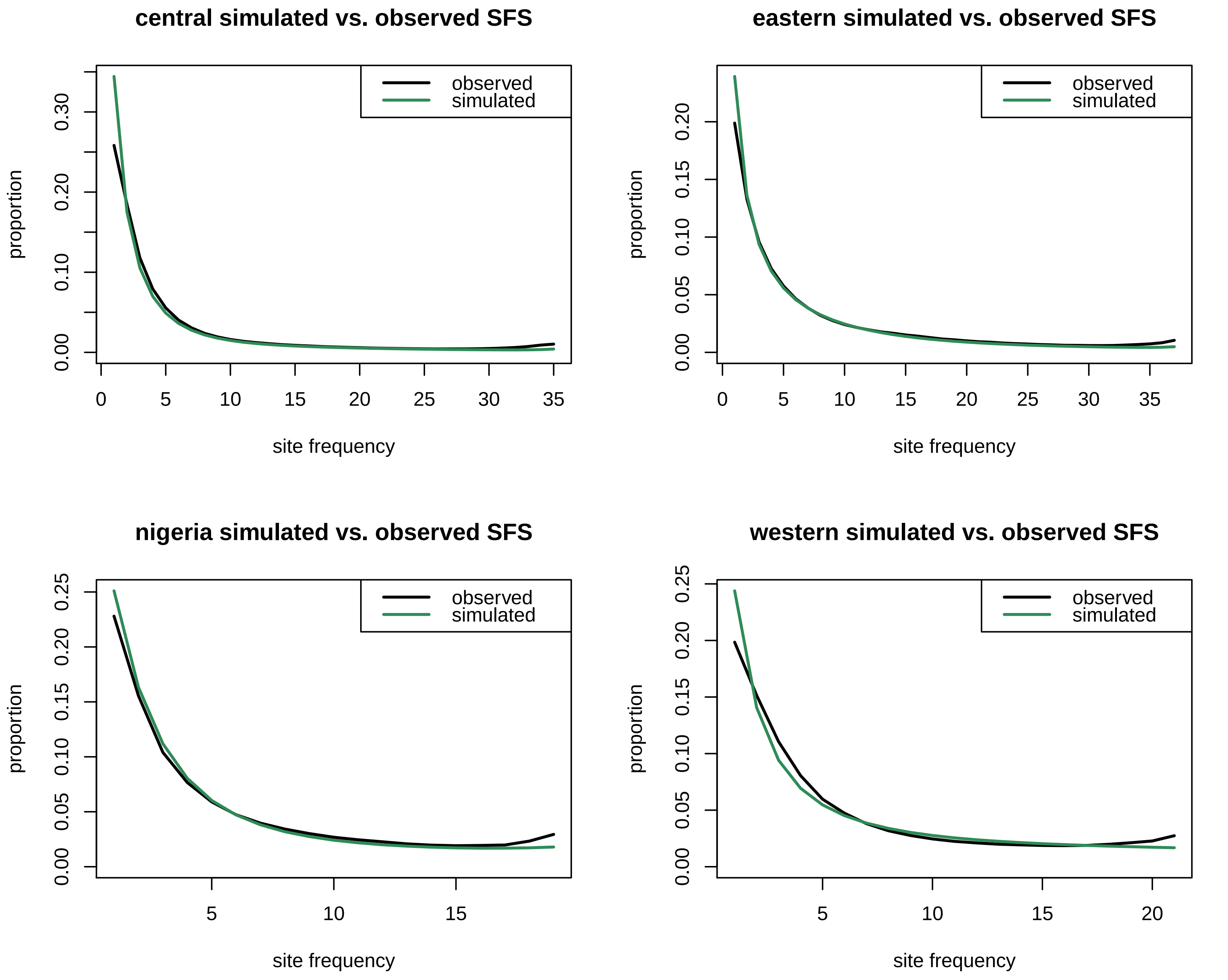
central simulated vs. observed SFS
eastern simulated vs. observed SFS
nigeria simulated vs. observed SFS
western simulated vs. observed SFS
observed
simulated
proportion
site frequency
0.00
0.05
0.10
0.15
0.20
0.25
0.30
0
5
10
15
20
25
30
35
